# Methods for Imaging Renin-Synthesizing, -Storing, and -Secreting Cells

**DOI:** 10.4061/2010/298747

**Published:** 2009-12-09

**Authors:** Daniel Casellas

**Affiliations:** Groupe Rein et Hypertension (EA3127), Institut Universitaire de Recherche Clinique, 641 Avenue du Doyen Giraud, 34093 Montpellier Cédex 5, France

## Abstract

Renin-producing cells have been the object of intense research efforts for the past fifty years within the field of hypertension. Two decades ago, research focused on the concept and characterization of the intrarenal renin-angiotensin system. Early morphological studies led to the concept of the juxtaglomerular apparatus, a minute organ that links tubulovascular structures and function at the single nephron level. The kidney, thus, appears as a highly “topological organ” in which anatomy and function are intimately linked. This point is reflected by a concurrent and constant development of functional and structural approaches. After summarizing our current knowledge about renin cells and their distribution along the renal vascular tree, particularly along glomerular afferent arterioles, we reviewed a variety of imaging techniques that permit a fine characterization of renin synthesis, storage, and release at the single-arteriolar, -cell, or -granule level. Powerful tools such as multiphoton microscopy and transgenesis bear the promises of future developments of the field.

## 1. Renin Cells in the Context of the Juxtaglomerular Apparatus (JGA) and Hypertension

Ever since Golgi published his seminal microdissection studies [1] on mammalian nephrons, our understanding of the JGA has emerged from a tight complementarity between anatomical and functional approaches [2–4]. The recent and fast development of digital confocal fluorescence microscopy [5–7] has considerably reduced the gap between structure and function. Such evolution can be illustrated by the recent “two-photon” fluorescence imaging of both cell structures, intracellular calcium signaling, and an array of regulatory mechanisms in live isolated-perfused JGA [8, 9].

Our knowledge about the nervous, tubular, and vascular structures that constitute a functional JGA unit was obtained by a variety of morphological techniques. They range from transmission electron microscopy (TEM, [3, 10–16]), scanning electron microscopy (SEM, [17–23]), histochemistry [24–27], fluorescence microscopy [6, 28–32], and immunohistochemistry [10, 25, 33–38], to atomic force microscopy [39]. Functional studies were carried out both in vivo and in vitro. They encompass the entire anatomical spectrum from whole kidney to single cell levels. They bring additional layers of complexity into our picture of the JGA with the tubuloglomerular feedback mechanism [40, 41], and cell signaling mechanisms in vessels and tubules [41–51].

The identification of cells with a synthetic (i.e., endocrine) phenotype along the afferent arteriole in close contact to nerve terminals, and located at the glomerular vascular pole near the macula densa led Goormaghtigh, seventy years ago, to the crucial concept that the JGA was a “neuro-myo-endocrine” organ (for historical account see: [10, 24, 52]). These granulated or epithelioid cells were later found to synthesize, store, and release renin [2–4, 10, 44, 53–55], a key enzyme of the renin-angiotensin system, and a key physiological player in the integrative control of blood pressure, glomerular hemodynamics, and in the homeostasis of sodium and electrolytes [41]. A recent review [56] on “renal vascular dysfunction in hypertension” fully confirms these views within the context of hypertension.

At this point, it is important to note that epithelioid cells are specialized smooth muscle cells present along the arteriovenous anastomoses of the rabbit ear vasculature [57]. Epithelioid cells are thus unlikely to be unique to the renal vasculature. Furthermore, renin-producing cells were also found in the media of the abdominal aorta in adult mice [58]. Immunoreactive renin was found in the cytoplasm of media cells in small blood vessels of human pulmonary tumors [59], and in a derived human tumor cell line, CaLu-6, that expresses the human renin gene endogenously [60, 61]. Interstitial fibroblast-like cells were found to express a complete renin-angiotensin system in a murine model of renal fibrosis [62].

Recent studies elegantly showed that renin cells differentiate in situ from progenitors scattered within the mesenchyme of the fetal kidney [25]. Subsequently, they colonize the early renal vasculature from arcuate arteries to afferent arterioles [63]. Most renin cells later adopt a smooth muscle cell phenotype [25, 64]. At early developmental stages, renin cells are associated with the development and branching patterns of the preglomerular vessels [34, 65], and with renal nerves [66]. However, it remains unknown whether renal nerves provide guidance cues and coordinate renal vascular branching, as it has recently been shown in the developing skin vasculature of mice [67], or more generally discussed in a recent review [68].

In the adult kidney, both renin cells and contractile smooth muscle cells populate the media of the afferent arteriole [10]. Under basal conditions, renin cells occupy a short (i.e., 20–40 *μ*m) arteriolar segment near the glomerular vascular pole [10, 36]. Under conditions known to stimulate renin synthesis (e.g., angiotensin I converting-enzyme inhibition, or low sodium diet, [69–71]), new renin-producing cells are recruited via the so-called “metaplastic transformation” of preexisting smooth muscle cells [10, 72–75]. Furthermore, renin cells may exhibit an array of ultrastructurally well-defined phenotypes from fully differentiated cells, at the tip of afferent arterioles, to “intermediate” cells which retain some characteristics of smooth muscle cells [10]. It is interesting to note that within a few days in primary culture, vascular smooth muscle cells shift from a contractile to an epithelioid (i.e., a synthetic) phenotype and loose their contractile proteins [76]. To the best of our knowledge, however, no renin production has been reported in these epithelioid cells in culture. Therefore, the “metaplastic” process leading to renin production appears as a characteristic of renal vascular smooth muscle cells. Alternatively, recent studies on renin cell lineage demonstrate that metaplasia affects cells that momentarily expressed renin during the early phase of the renal vascular development and later transformed into smooth muscle cells [25, 77].

The purpose of the present article is to review a series of techniques that permit the microscopic visualization of renin cells. These techniques can be incorporated into a variety of experimental designs as some of them can be used in live cells (i.e., use of vital dyes), whereas others require tissue processing. Immunohistochemical techniques which allow the assessment of renin cell distribution along renal vessels at a whole-kidney scale will be treated with more details, and illustrated.

The amount of intracellular renin represents a dynamic equilibrium between renin production/capture-storage, and release. As a logical complement, we will also examine techniques that permit the visualization of renin secretion and renin gene expression at the single cell level, though most of the time, these approaches involve enzymatic cell dispersion and a complete disruption of renal anatomy. As a final remark, it was beyond our scope to provide step-by-step protocols and the reader will be referred to the relevant literature for practical details.

## 2. Techniques for Visualization of Cellular Renin

### 2.1. Vital Dyes, Neutral Red, and Quinacrine

Histochemistry was developed early by microscopists. It provides chemical recipes to differentially stain cell organelles. The price to pay for its simplicity and directness is a limited specificity. This limitation can nevertheless be compensated for by using antibody-based approaches in a complementary way. Use of vital dyes is thus far the most direct way to stain intracellular renin granules.

Neutral red (2% solution in saline) accumulates in 1-2 hours within renin granules after its intraperitoneal injection, and subsequent observation is carried out on paraffin sections [24]. Direct cellular uptake of neutral red was recently used to highlight potentially renin-producing cells in the fetal renal mesenchyme [25]. Importantly, it was found that only 15% of neutral red-stained cells contained renin [25]. Of notice, use of neutral red uptake/release was instrumental in documenting stretch-induced secretion of atrial natriuretic peptide in single, isolated rat atrial myocytes [78].

The fluorescent dye quinacrine hydrochloride (Sigma) accumulates within 1 hour within renin granules after its intravenous administration (1 mg/kg), and can be imaged on paraffin sections [28]. This approach is functionally relevant since opposite changes in renin cell granularity and plasmatic levels of C^14^-labeled quinacrine were documented in circumstances associated with high renin secretion rates (i.e., hemorrhage and ischemia, [29]). In order to visualize both renin cell distribution and reactivity in live afferent arterioles, we recently incorporated quinacrine labeling into the in vitro blood-perfused juxtamedullary nephron preparation [79, 80]. Recently, using isolated-perfused rabbit glomeruli and the high resolving power of two-photon confocal microscopy, Peti-Peterdi et al. [81] were able to record, for the first time, the exocytosis of a single renin granule. Some potential limitations of the latter approach evoked in a recent review [42] will be dealt with in Section 3.6.

### 2.2. Immunohistochemistry and Photonic Microscopy

**Figure 1:**
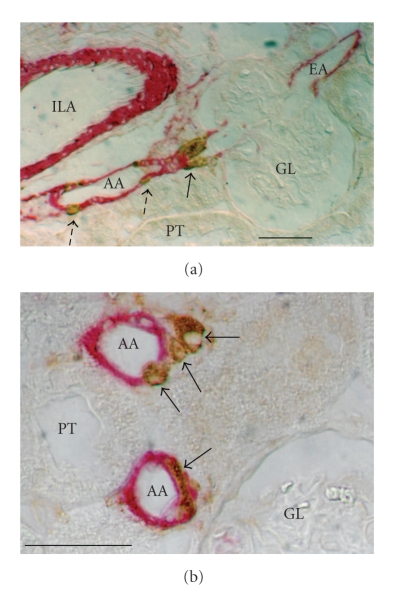
Microscopic views of paraffin kidney sections from a rat chronically treated with the converting enzyme inhibitor captopril (30 mg per day for 7 weeks per os; Sigma, St Louis, MO). Sections were double-stained against *α*-smooth muscle actin (red label, monoclonal anti-*α*-SM actin, clone # 1A4, 1 : 1000 dilution, Sigma), and against renin (brown label, polyclonal anti-rat renin antibody, ref. # 936, 1 : 10,000 dilution, gift from T. Inagami, Vanderbuilt University, Nashville, TN), using Dako En Vision Doublestain System (Ref. K1395, Dako A/S, Glostrup, Denmark). (a) A juxtamedullary glomerulus (GL) with its afferent (AA) and efferent arterioles (EA), close to an interlobular artery (ILA), and proximal tubules (PT). Renin cells accumulate near the glomerular vascular pole (single arrow) and other renin cells were recruited upstream along the afferent arteriole (dashed arrows). (b**)** Enlarged view of two transversally cut afferent arterioles (AA) near a glomerulus (GL). Arrows point to individual granulated renin cells (brown label) that exhibit a variety of topologies with respect to smooth muscle cells (red label). The renin cell in the lower arteriole is included within the arteriolar wall between smooth muscle cell processes. In the upper arteriole, one renin cell (lower arrow) is part of the arteriolar wall, whereas the upper two renin cells are located on top of the smooth muscle cell layer. Regarding the latter two observations, it is interesting to note that recent studies in connexin 40-deficient mice [82] unveiled a “topological escape” of renin-cells away from afferent arterioles towards the extraglomerular mesangium. Bars: 50 *μ*m.

Immunohistochemistry has become very popular due to the commercial availability of many antibodies, and easy-to-use staining kits for single- or double-immunolabeling (e.g., staining kits fromVector Laboratories, Burlingame, CA, or from Dako A/S, Glostrup, Denmark). This approach allows the visualization of renin cell distribution in whole kidney sections which preserves topological characteristics. Classic tissue processing involves perfusion-fixation of the kidney, paraffin embedding, sectioning, antigen retrieval, and immunoperoxidase procedures (details can be found in [33, 83]). Examples of immunostaining of renin combined with that of *α*-smooth muscle actin to highlight blood vessels are given in Figure 1(a). Complex topologies of renin and smooth muscle cells can thus be revealed on tissue sections (Figure 1(b)).

Tissue sectioning, however, limits the researcher's ability to get an integrated view of the distribution of renin cells throughout the preglomerular vasculature. Recent developments in digital image processing [84] would theoretically facilitate reconstruction of renin cell distribution from serial sectioning, but this approach remains time consuming. In fact, similar approach was recently performed on immunostained serial paraffin sections and superbly illustrated the “development of renin expression in the mouse kidney” [63]. More recently, [85] similar reconstruction approach was performed in transgenic mice to assess the impact of altered cyclic AMP pathway on the developmental regulations of vascular renal renin expression. Similarly, studies assessed the impact of connexin 40 in Cx40-deficient transgenic mice on vascular renin cell distribution [82]. Furthermore, as these studies [63, 82, 85] use serial paraffin sections, and since “standard” confocal microscopy currently allows the imaging of three fluorophores [86], additional information to characterize the surrounding renal parenchyma or intestitium (e.g., collagen IV) could be obtained and processed three-dimentionally by using an appropriate additional immunostaining.

**Figure 2:**
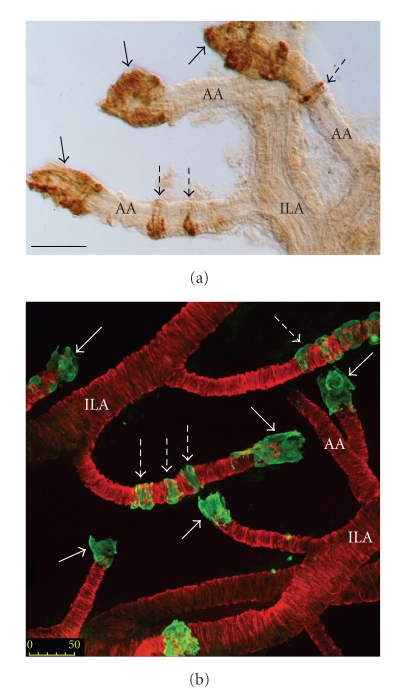
Portions of the renal preglomerular vasculature comprised of an interlobular artery (ILA) and afferent arterioles (AA) were isolated after HCl hydrolysis in rats chronically treated with captopril (see legend to Figure 1). Renin cells are localized to the AA tip (single arrows); captopril treatment also recruits renin cells upstream (dashed arrows). (a) Single labeling against renin (see Figure 1 legend) with ABC peroxidase kit (brown label, Vector Laboratories, Burlingame, CA). Recruited renin cells are spindle-shaped as smooth muscle cells, whereas renin cells at the tip of AA adopt a more globular shape. (b**)** Double labeling against renin (green label, fluorescein isothiocyanate-labeled secondary anti body) and against *α*-SM actin (red label, tetramethylrhodamine-labeled secondary antibody); for primary antibodies see legend to Figure 1. The image was generated as previously detailed [86] using a confocal microscope (Biorad MRC1024, Bio-Rad Life Science Research, Hercules, CA) by projecting 52 serial optical sections. Bars: 50 *μ*m.

In a more straightforward way, we designed a technique that associates isolation of preglomerular vascular trees after HCl hydrolysis, and immunostaining for renin [33]. Dissection of HCl-macerated kidneys yields long segments of the preglomerular vasculature with preserved spatial geometry. Cells full of renin granules refract incident light differently than smooth muscle cells do and can thus be spotted under a dissecting microscope without staining. After immunostaining, vascular distribution of renin cells and their status (i.e., “intermediate” or fully differentiated cells) can be determined precisely under the microscope (Figure 2(a)). Interestingly, an excellent correlation (*r* = 0.84) was found between the relative frequency of afferent arterioles with renin cells recruited along their mid-portion, and renal renin activity [69]. Our approach has been instrumental in various subsequent studies [34, 43, 58, 87]. As illustrated in Figure 2(b), the use of confocal microscopy, with its optical sectioning capability, allows a detailed study of the morphology of individual renin cells within their vascular environment.

### 2.3. Combining Photonic Microscopy, Microdissected Afferent Arterioles, Primary Cultures of Isolated Juxtaglomerular Renin Cells, and Renin Assay

Several recent studies [88, 89] took advantage of previously developped techniques [90] to obtain primary cell cultures of mouse juxtaglomerular renin-cell cultures, a radioimmunoassay of renin synthesis and renin secretion from these cultures, and combined it with the optical power of fluorescence confocal microscopy. These authors [88, 89] demonstrated colocalization of adenylyl cyclase isoform V and renin within granules and provided important clues on signaling mechanisms and proteins involved in renin release. Previous studies approached the role of adenosine on renin release [91] based on similar renin assay [90] and using isolated superfused juxtaglomerular cells from rat kidneys. Other studies [60] combining primary cell cultures of rat juxtaglomerular cells, human tumor cell line (CaLu-6, [61]), renin enzymatic assay, and a variety of optical techniques provided important clues about the existence of a biomechanical coupling in renin-releasing cells (i.e., the so-called “intrarenal baroreceptor”, [92]). Previously, using microdissected rabbit afferent arterioles with or without attached tubules containing the macula densa segment and a renin radioimmunoassay [93] permitted important advances in our understanding of renin release (reviewed in [42, 94]).

### 2.4. Transmission Electron Microscopy (TEM)

TEM is the method of choice to explore cell ultrastructure in tiny volumes of tissue. TEM methodologies were implemented during the fifties. They involve strong aldehyde fixation for proper structural preservation, incorporation of heavy metals to generate contrast, and ultramicrotomy for ultra-thin sectioning. We owe this technique most of our current knowledge about the ultrastructure of renin cells, renin granules, and their cellular processing [2, 3, 10, 53, 95, 96]. Protein antigenicity may nevertheless be preserved by milder fixation, though at the cost of structural definition. Specific antibodies and the protein A-gold technique can then be used to detect renin and/or other proteins within granules (for details and illustrations see [10, 97, 98]).

### 2.5. Scanning Electron Microscopy (SEM)

SEM allows the observation of surface details in whole kidney sections with unprecedented depth of field, and offers a wide range of magnifications (e.g., [17–19]). The development of high resolution field-emission SEM and a variety of cytoplasmic extraction and immunostaining procedures allow a unique, three-dimensional imaging of intracellular organelles (spectacular illustrations can be found in: [99, 100]). Despite such imaging potential, few studies, so far, have attempted to visualize intracellular renin granules by SEM (e.g., [20]).

## 3. Visualization of Single-Arteriolar, Single-Cell, or Single-Granule Renin Secretion

### 3.1. The Reverse Hemolytic Plaque Assay

An adaptation of the reverse hemolytic plaque assay allows a microscopic evaluation of renin release at the single cell level [101]. This elegant technique is performed on renal cell suspensions in vitro. Released renin is captured by a specific anti-renin antibody. The renin-antibody complex will then bind to protein A-conjugated sheep erythrocytes which will in turn be hemolyzed by complement attack. Renin secretion is visualized in a semiquantitative way as a circle of hemolyzed erythrocytes around individual renin-producing cells (complete technical description can be found in: [97, 101]).

### 3.2. The Cell Blot Assay

A very elegant method was developed to image single cell protein release, the so-called cell blot assay [102]. Secreted proteins are irreversibly captured by a polyvinyldiene difluoride transfer membrane on top of which cell suspensions are deposited. Proteins are then recognized by an appropriate primary antibody, and visualized with a secondary antibody as peroxidase [102] or fluorescent halos around secreting cells [103]. This technique is yet to be used with renin cells but might constitute a simpler alternative to the reverse hemolytic plaque assay (Section 3.1).

### 3.3. Video-Enhanced Differential Interference-Contrast Microscopy

Differential interference-contrast (DIC) microscopy allows thin optical sectioning and detailed imaging of live cells without staining (e.g., [104]). Combined with video-enhancement procedures, DIC microscopy resolves submicrometer details [105]. This approach has been successfully applied to the direct study of movement and exocytosis of single secretory granules in intact tissue [106] or in dispersed cells [107]. Its application in renin cells is yet to be done.

### 3.4. Evanescent Wave Fluorescence Microscopy

Evanescent-wave microscopy is based upon the total internal reflection of a laser light beam directed at the interface between a glass coverslip and a cell tightly attached to it. Total internal reflection generates an evanescent light field 100–300 nm thick that will illuminate any fluorescently tagged particle that penetrates it. This very powerful optical approach has been used in neurons [108], chromaffin [109], or pancreatic *β* cells [110, 111] to document single-vesicle or single-granule approach, docking and exocytosis in real time, optically sorting dense-core vesicles in chromaffin cells according to their age [112], and for single molecule imaging within cells [113]. As techniques are available to isolate and culture granulated renin cells (e.g., [60]), further developments may be expected for renin cells in this field.

### 3.5. Ultramicro-Radioimmunoassay of Renin Concentration/Whole-Cell Patch-Clamp Technique

The “quantal” nature of renin release [114], and its relationship with fusion of renin granules with cell membrane [96] were first demonstrated by combining a unique ultramicroradioimmunoassay of renin concentration [115], superfusion of single isolated rat afferent arterioles, and TEM of renin granules in the same superfused single isolated rat afferent arterioles [96]. The same renin assay [115] was later combined with a newly developed isolated perfused rabbit macula densa preparation [104]. For the first time [54], the inverse relationship between tubular fluid sodium chloride concentration at the macula densa and renin release rate of the afferent arteriole was demonstrated. More recently whole-cell patch-clamp techniques were successfully used to demonstrate exocytosis and endocytosis in single mouse juxtaglomerular cells in relationship with known stimuli or inhibitors of renin release [42, 44, 116].

### 3.6. Two-Photon Confocal Microscopy

As previously mentioned (Section 2.1), the exocytosis of a single renin granule was documented using two-photon confocal microscopy and the in vitro microperfused rabbit afferent arterioles whose renin was stained with the acidotropic fluorophore quinacrine [81]. In the same study, renin granules were stained by perfusion with an acidotropic lysosomal fluorophores, Lyso Tracker-Red (Molecular Probes), and increases in renin activity resulting from renin granule release were probed within the perivascular space with EDANS (Molecular Probes), allowing simultaneous imaging of renin release and activity in a single perfused afferent arteriole [81]. Despite the unprecedented imaging capacity of multiphoton imaging and the multiple possibilities offered to functional approaches in a live, integrated nephro-vascular unit (reviewed recently in [8, 117]), one must underline some specific limits to the observation of the behaviour of renin cells and renin granules. Though careful validations were performed (i.e., colocalization of quinacrine and renin immunolabelling within granules in mouse kidney sections, granule release occurring as burst or quantal release, release occurring in response to isoproterenol, or decrease in arteriolar perfusion pressure, [81]), these studies rely on the assumption that surrogate molecules (i.e., quinacrine, Lyso Tracker-Red, [81]) rather than renin itself are observed. Remarquably, too, an estimated renin release affecting 40% of cell granules disappearing in 10 minutes [81] is substantially higher than 1-2% release rates obtained in dissected afferent arterioles in rats [96, 114] or rabbits [93] based respectively on renin ultramicro-radioimmunoassay or radioimmunoassay. Along these lines, a “relative rarity of exocytosis events” was previously noted in TEM studies in mice renal cortical slices [95] and was recently underlined in a review on renin release [42]. Furthermore, and still poorly appreciated in the renal microcirculation field, interactions between light and fluorophores may generate heat and photochemical processes that may affect protein synthesis in live specimens. The so-called “light-dye” or “laser-dye” effects locally and/or temporarily modify endothelial cell function (e.g., [118, 119]), and the impact of confocal microscopy on cell calcium handling and cell death has recently been emphasized in bovine chondrocyte cultures [120]. Further studies seem therefore warranted to settle these issues linked to “classic” fluorescence, mono-, or multiphoton imaging in live tissues.

## 4. Visualization of Renin Gene Expression

### 4.1. In Situ Hybridization

Since circulating renin may be captured by vascular cells [121], it is important to determine, by in situ hybridization, whether renin synthesis occurs in cells that stock renin. Protocols based on the use of radioactive [60, 122] or nonradioactive, digoxigenin-labeled riboprobes [123] are currently available to visualize cellular renin mRNA on kidney sections or cultured cells. One must note that in situ hybridization was combined with the reverse hemolytic plaque assay (i.e., Section 3.1) to visualize both gene expression and peptide secretion in single cells [124].

### 4.2. Single-Cell Reverse Transcription-Polymerase Chain Reaction Technique

The detection of specific mRNAs within a single-cell is made possible by the reverse transcription-polymerase chain reaction technique initially developed for cultured neurons [125]. This approach requires cell isolation. It has been successfully performed in glomerular podocytes individually aspirated with a micropipet [126], in freshly dispersed smooth muscle cells [52, 127], in renal epithelial cells [128], and in renin cells aspirated from embryonic kidneys [25]. Detection of single cell transcripts is performed on agarose gels. Using the so-called in situ reverse transcription polymerase chain reaction technique [129], transcripts can be visualized within individual cells with digoxigenin staining [130].

### 4.3. Laser Capture Microdissection

A laser capture microdissection technique was recently developed from Ashkin's “optical tweezers” [131]. Under microscopic observation, small cell samples or single cells can be dissected with a sharp laser beam from precise areas of thin cryostat or paraffin sections, and optically catapulted into a test tube. Anatomical localization is facilitated by hematoxylin or immunofluorescence staining. In association with single-cell reverse transcription-polymerase chain reaction technique, this technique opens new avenues in the study of spatial distribution of gene expression [132–135].

### 4.4. Transgenic Mice

**Figure 3:**
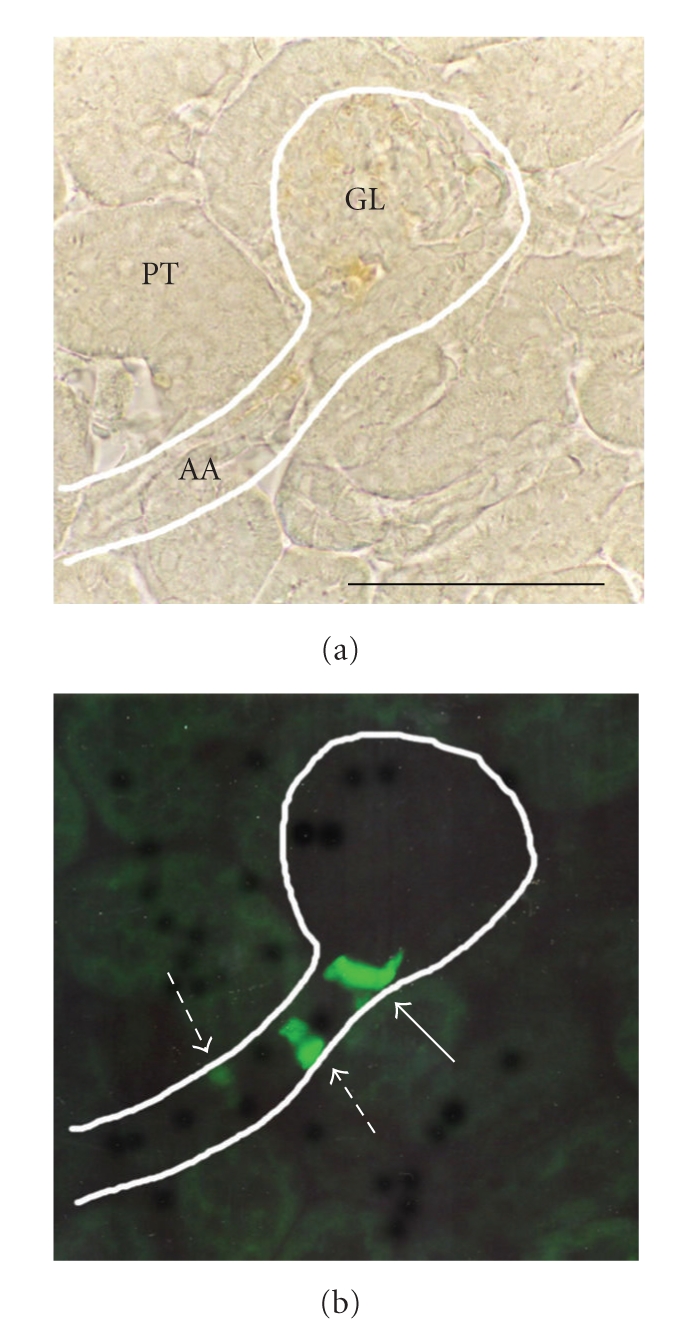
Light micrographs of a cryostat kidney section. Renal tissue was obtained from a transgenic heterozygous (Ren 1^d^-GFP/^+^) adult mouse in which green fluorescent protein (GFP) was placed under the control of the Ren 1^d^ renin locus by homologous recombination [25, 72]. Renin synthesis was stimulated by a 7-day-treatment with the angiotensin II type-1 receptor antagonist, losartan (25 mg/kg per day). (a**)** brightfield view showing the outline of an afferent arteriole (AA) and a glomerulus (GL) among proximal tubules (PT). (b) fluorescence view of the same field (white outline) showing green GFP signal from renin-synthesizing cells at the glomerular vascular pole (single arrow), and from upstream recruited cells (dashed arrows). Bar: 100 *μ*m.

Molecular biology and transgenic approaches (e.g., homologous recombination) have introduced new ways to visualize renin gene expression and its regulation in vivo during development, and during adulthood. Various transgenic mice have been recently generated. Green fluorescent protein (GFP) or LacZ reporter genes are fused to the appropriate promoter sequences and allow to follow with high fidelity renin gene expression in the renal vasculature. Imaging may either be performed directly by fluorescence microscopy of live tissues [25, 72, 136], or *β*-galactosidase activity is detected by X-gal staining after tissue processing [58].  Figure 3 illustrates the afferent arteriolar distribution of cells expressing the Ren1^d^ gene as revealed by the direct fluorescence study of GFP-expressing cells. In this example, renin synthesis was boosted by a one-week treatment with losartan in cells localized at the glomerular vascular pole (Figure 3, single arrow) and in cells recruited upstream (Figure 3, dashed arrows). These transgenic strains will be crucial for future elucidation of the molecular determinants of gene expression patterns in vivo. 

## 5. Conclusions

A variety of techniques and procedures are now in the researcher's toolbox to visualize renin cells, and more will emerge from researchers' and engineers' relentless inventiveness. Renin gene expression, renin storage/processing, and renin release are three basic aspects of cellular renin processing that can be imaged. Some techniques were listed (i.e., Sections 2.4, 3.2, 3.3, 3.4, 4.3) because they would deserve an application to renin cells. For instance, they have the potential to provide insights into the processing and exocytosis of single renin granules, and may help to approach the “calcium paradox” and the role played by myofilaments in “intermediate” renin cells [95, 137]. Several of these techniques may be or have been combined to assess synthesis, storage, and release at the single cell level and have provided important knowledge on cell pathways leading to renin release (recently reviewed in [42]). The vascular topology or renin cells can be well preserved in various approaches dealing with renin storage and renin gene expression. In the well-studied rat species, it was observed that sodium depletion leads to the recruitment of renin-containing cells along afferent arterioles, whereas renin gene expression, as probed by in situ hybridization, increased near glomerular vascular poles only [73]. This spatial discrepancy between gene expression and protein distribution was interpreted as indicative of either renin uptake, or ephemeral expression of the renin gene [70]. As a result, the distribution of renin-positive cells along preglomerular vessels will not always reflect renin gene expression in the rat, as is the case during inhibition of the renin-angiotensin system [72, 73]. Future studies will help unfolding the complex nature of the spatio-temporal relationships between renin gene expression and protein processing. The story becomes more complex in some mice strains which may have two cooperative renin genes [72]. The development of transgenic mice whose fluorescent reporter gene accurately mimics the spatio-temporal expression patterns of the endogenous renin gene(s), provides a spectacular and promising new tool for the direct study of renin cells and JGA in situ [72, 136].

As a final remark, technical choices will always be served by a thorough critical review of inherent, though occasionally unsuspected limits, sometimes better appreciated in a nearby field of research which then becomes a “source” for fruitful new applications.

## References

[1] Golgi C (1889). Annotazioni intorno all’ Istologia die reni dell’ uomo e di altri mammiferi e sull’ Istogenesis die canalicoli oriniferi. *Atti della Reale Accademia dei Lincei, Rendiconti*.

[2] Barajas L (1979). Anatomy of the juxtaglomerular apparatus. *American Journal of Physiology*.

[3] Barajas L (1981). The juxtaglomerular apparatus: anatomical considerations in feedback control of glomerular filtration rate. *Federation Proceedings*.

[4] Barajas L, Salido EC, Liu L, Powers KV, Laragh JH, Brenner BM (1995). The juxtaglomerular apparatus: a morphologic perspective. *Hypertension: Pathophysiology, Diagnosis, and Management*.

[5] Clendenon JL, Phillips CL, Sandoval RM, Fang S, Dunn KW (2002). Voxx: a PC-based, near real-time volume rendering system for biological microscopy. *American Journal of Physiology*.

[6] Kwon O, Phillips CL, Molitoris BA (2002). Ischemia induces alterations in actin filaments in renal vascular smooth muscle cells. *American Journal of Physiology*.

[7] Saino T, Matsuura M, Satoh Y-I (2002). Application of real-time confocal microscopy to intracellular calcium ion dynamics in rat arterioles. *Histochemistry and Cell Biology*.

[8] Peti-Peterdi J, Toma I, Sipos A, Vargas SL (2009). Multiphoton imaging of renal regulatory mechanisms. *Physiology*.

[9] Peti-Peterdi J, Morishima S, Bell PD, Okada Y (2002). Two-photon excitation fluorescence imaging of the living juxtaglomerular apparatus. *American Journal of Physiology*.

[10] Taugner R, Hackenthal E (1989). *The Juxtaglomerular Apparatus*.

[11] Barajas L, Latta H (1963). A three-dimensional study of the juxtaglomerular apparatus in the rat. Light and electron
microscopic observations. *Laboratory Investigation*.

[12] Elger M, Sakai T, Kriz W, Beck F, Christ B, Marani E, Sano Y, Zilles K (1998). The vascular pole of the renal glomerulus of rat. *Advances in Anatomy Embryology and Cell Biology*.

[13] Inkyo-Hayasaka K, Sakai T, Kobayashi N, Shirato I, Tomino Y (1996). Three-dimensional analysis of the whole mesangium in the rat. *Kidney International*.

[14] Jones DB, Barber Mueller C, Menefee M (1962). The cellular and extracellular morphology of the glomerular stalk. *American Journal of Pathology*.

[15] Kaissling B, Kriz W (1982). Variability of intercellular spaces between macula densa cells: a transmission electron microscopic study in rabbits and rats. *Kidney International*.

[16] Latta H, Maunsbach AB, Madden SC (1960). The centrolobular region of the renal glomerulus studied by electron microscopy. *Journal of Ultrasructure Research*.

[17] Casellas D (1986). A method for scanning electron microscopic observation of glomerular vascular poles in rat
kidneys. *Journal of Electron Microscopy Technology*.

[18] Gattone VH, Luft FC, Evan AP (1984). Renal afferent and efferent arterioles of the rabbit. *American Journal of Physiology*.

[19] Jones DB (1985). Methods in laboratory investigation. Enzymatic dissection of the glomerulus. *Laboratory Investigation*.

[20] Isler H, Krstić R (1981). Scanning electron microscopy of the juxtaglomerular apparatus in the freeze-fractured rat kidney. *Archivum Histologicum Japonicum*.

[21] Carlson EC, Hinds D (1983). A topographical (SEM) analysis of acellular glomerular mesangial matrix in situ. *Journal of Ultrastructure Research*.

[22] Makino H, Hironaka K, Shikata K (1994). Mesangial matrices act as mesangial channels to the juxtaglomerular zone. Tracer and high-resolution scanning electron-microscopic study. *Nephron*.

[23] Takahashi-Iwanaga H (1992). Three-dimensional visualization of renal cells by NaOH maceration. *Archives of Histology and Cytology*.

[24] Harada K (1954). Histochemical studies of the juxta glomerular apparatus. *Revue Belge de Pathologie et de Medecine Experimentale*.

[25] Sequeira López MLS, Pentz ES, Robert B, Abrahamson DR, Gomez RA (2001). Embryonic origin and lineage of juxtaglomerular cells. *American Journal of Physiology*.

[26] Faarup P (1965). On the morphology of the juxtaglomerular apparatus. *Acta Anatomica*.

[27] Sato T, Trump BF (1982). A simple method of staining juxtaglomerular granules in the rat kidney for high resolution light microscopy. *Stain Technology*.

[28] Ålund M, Olson L (1979). Quinacrine affinity of endocrine cell systems containing dense core vesicles as visualized by fluorescence microscopy. *Cell and Tissue Research*.

[29] Ålund M (1980). Juxtaglomerular cell activity during hemorrhage and ischemia as revealed by quinacrine histofluorescence. *Acta Physiologica Scandinavica*.

[30] Barajas L (1978). Innervation of the renal cortex. *Federation Proceedings*.

[31] Kobayashi N, Sakai T (1993). Heterogeneity in the distribution of actin filaments in the endothelial cells of arteries and arterioles in the rat kidney. *European Journal of Cell Biology*.

[32] Zwi LJ, Sharada K, Ormrod DJ, Edgar SG (1999). Visualization of blood vessels without prior perfusion. *Microvascular Research*.

[33] Casellas D, Dupont M, Kaskel FJ, Inagami T, Moore LC (1993). Direct visualization of renin-cell distribution in preglomerular vascular trees dissected from rat kidney. *American Journal of Physiology*.

[34] Reddi V, Zaglul A, Pentz ES, Gomez RA (1998). Renin-expressing cells are associated with branching of the developing kidney vasculature. *Journal of the American Society of Nephrology*.

[35] Carey AV, Carey RM, Gomez RA (1992). Expression of *α*-smooth muscle actin in the developing kidney vasculature. *Hypertension*.

[36] Casellas D, Taugner R (1986). Renin status of the afferent arteriole and ultrastructure of the juxtaglomerular apparatus in “superficial” juxtamedullary nephrons from rats. *Renal Physiology*.

[37] Liu L, Barajas L (1993). The rat renal nerves during development. *Anatomy and Embryology*.

[38] Tanaka K, Fukudome H (1991). Three-dimensional organization of the Golgi complex observed by scanning electron microscopy. *Journal of Electron Microscopy Technique*.

[39] Henderson RM, Oberleithner H (2000). Pushing, pulling, dragging, and vibrating renal epithelia by using atomic force microscopy. *American Journal of Physiology*.

[40] Casellas D, Moore LC (1990). Autoregulation and tubuloglomerular feedback in juxtamedullary glomerular arterioles. *American Journal of Physiology*.

[41] Navar LG, Inscho EW, Majid DSA, Imig JD, Harrison-Bernard LM, Mitchell KD (1996). Paracrine regulation of the renal microcirculation. *Physiological Reviews*.

[42] Schweda F, Friis U, Wagner C, Skøtt O, Kurtz A (2007). Renin release. *Physiology*.

[43] Hansen PB, Jensen BL, Andreasen D, Skøtt O (2001). Differential expression of T- and L-type voltage-dependent calcium channels in renal resistance vessels. *Circulation Research*.

[44] Friis UG, Jensen BL, Aas JK, Skøtt O (1999). Direct demonstration of exocytosis and endocytosis in single mouse juxtaglomerular cells. *Circulation Research*.

[45] Bell PD, Lapointe JY, Peti-Peterdi J (2003). Macula densa cell signaling. *Annual Review of Physiology*.

[46] Casellas D, Carmines PK (1996). Control of the renal microcirculation: cellular and integrative perspectives. *Current Opinion in Nephrology and Hypertension*.

[47] Chatziantoniou C, Arendshorst WJ (1993). Angiotensin receptor sites in renal vasculature of rats developing genetic hypertension. *American Journal of Physiology*.

[48] Inscho EW, Mason MJ, Schroeder AC, Deichmann PC, Stiegler KD, Imig JD (1997). Agonist-induced calcium regulation in freshly isolated renal microvascular smooth muscle cells. *Journal of the American Society of Nephrology*.

[49] Kurtz A (1989). Cellular control of renin secretion. *Reviews of Physiology Biochemistry and Pharmacology*.

[50] Loutzenhiser K, Loutzenhiser R (2000). Angiotensin II-induced Ca^2+^ influx in renal afferent and efferent arterioles: differing roles of voltage-gated and store-operated Ca^2+^ entry. *Circulation Research*.

[51] Moore LC, Iijima K, Rich A, Casellas D, Goligorsky MS (1991). Communication of the tubuloglomerular feedback signal in the JGA. *Kidney International*.

[52] Thurau K (1982). The juxtaglomerular apparatus, 1925–1981. *Kidney International*.

[53] Rasch R, Jensen BL, Nyengaard JR, Skøtt O (1998). Quantitative changes in rat renin secretory granules after acute and chronic stimulation of the renin system. *Cell and Tissue Research*.

[54] Skøtt O, Briggs JP (1987). Direct demonstration of macula densa-mediated renin secretion. *Science*.

[55] Hackenthal E, Paul M, Ganten D, Taugner R (1990). Morphology, physiology, and molecular biology of renin secretion. *Physiological Reviews*.

[56] Ponnuchamy B, Khalil RA (2009). Cellular mediators of renal vascular dysfunction in hypertension. *American Journal of Physiology*.

[57] Gölfert F, Kasper M, van Eys GJJM, Funk RHW (1997). Cytoskeletal characterization of arteriovenous epithelioid cells. *Histochemistry and Cell Biology*.

[58] Fuchs S, Germain S, Philippe J, Corvol P, Pinet F (2002). Expression of renin in large arteries outside the kidney revealed by human renin promoter/LacZ transgenic mouse. *American Journal of Pathology*.

[59] Taylor GM, Cook HT, Sheffield EA, Hanson C, Peart WS (1988). Renin in blood vessels in human pulmonary tumors. An immunohistochemical and biochemical study. *American Journal of Pathology*.

[60] Carey RM, McGrath HE, Pentz ES, Gomez RA, Barrett PQ (1997). Biomechanical coupling in renin-releasing cells. *The Journal of Clinical Investigation*.

[61] Lang JA, Ying LI-H, Morris BJ, Sigmund CD (1996). Transcriptional and posttranscriptional mechanisms regulate human renin gene expression in Calu-6 cells. *American Journal of Physiology*.

[62] Okada H, Inoue T, Kanno Y (2002). Interstitial fibroblast-like cells express renin-angiotensin system components in a fibrosing murine kidney. *American Journal of Pathology*.

[63] Sauter A, Machura K, Neubauer B, Kurtz A, Wagner C (2008). Development of renin expression in the mouse kidney. *Kidney International*.

[64] Gomez RA, Norwood VF (1995). Developmental consequences of the renin-angiotensin system. *American Journal of Kidney Diseases*.

[65] Gomez RA, Pupilli C, Everett AD (1991). Molecular and cellular aspects of renin during kidney ontogeny. *Pediatric Nephrology*.

[66] Pupilli C, Gomez RA, Tuttle JB, Peach MJ, Carey RM (1991). Spatial association of renin-containing cells and nerve fibers in developing rat kidney. *Pediatric Nephrology*.

[67] Mukouyama Y-S, Shin D, Britsch S, Taniguchi M, Anderson DJ (2002). Sensory nerves determine the pattern of arterial differentiation and blood vessel branching in the skin. *Cell*.

[68] Horowitz A, Simons M (2008). Branching morphogenesis. *Circulation Research*.

[69] Casellas D, Dupont M, Bouriquet N, Moore LC, Artuso A, Mimran A (1994). Anatomic pairing of afferent arterioles and renin cell distribution in rat kidneys. *American Journal of Physiology*.

[70] Gomez RA, Chevalier RL, Carey RM, Peach MJ (1990). Molecular biology of the renal renin-angiotensin system. *Kidney International*.

[71] Juncos LA, Ito S, Nobiling R, Carretero OA (1992). Renin distribution in the rabbit renal microvasculature. *Hypertension*.

[72] Pentz ES, Sequeira López MLS, Kim H-S, Carretero O, Smithies O, Gomez RA (2001). Ren1^d^ and Ren2 cooperate to preserve homeostasis: evidence from mice expressing GFP in place of Ren1^d^. *Physiological Genomics*.

[73] Gomez RA, Chevalier RL, Everett AD (1990). Recruitment of renin gene-expressing cells in adult rat kidneys. *American Journal of Physiology*.

[74] Cantin M, Araujo-Nascimento MF, Benchimol S, Desormeaux Y (1977). Metaplasia of smooth muscle cells into juxtaglomerular cells in the juxtaglomerular apparatus, arteries, and arterioles of the ischemic (endocrine) kidney. An ultrastructual cytochemical and autoradiographic study. *American Journal of Pathology*.

[75] Gomez RA, Lynch KR, Chevalier RL (1988). Renin and angiotensinogen gene expression and intrarenal renin distribution during ACE inhibition. *American Journal of Physiology*.

[76] Thyberg J (2000). Differences in caveolae dynamics in vascular smooth muscle cells of different phenotypes. *Laboratory Investigation*.

[77] Sequeira López MLS, Pentz ES, Nomasa T, Smithies O, Gomez RA (2004). Renin cells are precursors for multiple cell types that switch to the renin phenotype when homeostasis is threatened. *Developmental Cell*.

[78] Ryu SY, Lee S-H, Isenberg G, Ho W-K, Earm YE (2002). Monitoring of ANP secretion from single atrial myocytes using densitometry. *Pflügers Archiv European Journal of Physiology*.

[79] Casellas D, Navar LG (1984). In vitro perfusion of juxtamedullary nephrons in rats. *American Journal of Physiology*.

[80] Casellas D, Carmines PK, Dupont M, Redon P, Moore LC (1990). Arteriolar renin and vascular effects of angiotensin II in juxtamedullary nephrons. *Kidney International*.

[81] Peti-Peterdi J, Fintha A, Fuson AL, Tousson A, Chow RH (2004). Real-time imaging of renin release in vitro. *American Journal of Physiology*.

[82] Kurtz L, Schweda F, de Wit C (2007). Lack of connexin 40 causes displacement of renin-producing cells from afferent arterioles to the extraglomerular mesangium. *Journal of the American Society of Nephrology*.

[83] Bribes E, Casellas P, Vidal H, Dussossoy D, Casellas D (2002). Peripheral benzodiazepine receptor mapping in rat kidney. Effects of angiotensin II-induced hypertension. *Journal of the American Society of Nephrology*.

[84] Zhai XY, Birn H, Jensen KB, Thomsen JS, Andreasen A, Christensen EI (2003). Digital three-dimensional reconstruction and ultrastructure of the mouse proximal tubule. *Journal of the American Society of Nephrology*.

[85] Neubauer B, Machura K, Chen M (2009). Development of vascular renin expression in the kidney critically depends on the cyclic AMP pathway. *American Journal of Physiology*.

[86] Darlot F, Artuso A, Lautredou-Audouy N, Casellas D (2008). Topology of Schwann cells and sympathetic innervation along preglomerular vessels: a confocal microscopic study in protein S100B/EGFP transgenic mice. *American Journal of Physiology*.

[87] Kim H-S, Maeda N, Oh GT, Fernandez LG, Gomez RA, Smithies O (1999). Homeostasis in mice with genetically decreased angiotensinogen is primarily by an increased number of renin-producing cells. *The Journal of Biological Chemistry*.

[88] Ortiz-Capisano MC, Ortiz PA, Harding P, Garvin JL, Beierwaltes WH (2007). Adenylyl cyclase isoform V mediates renin release from juxtaglomerular cells. *Hypertension*.

[89] Ortiz-Capisano MC, Ortiz PA, Harding P, Garvin JL, Beierwaltes WH (2007). Decreased intracellular calcium stimulates renin release via calcium-inhibitable adenylyl cyclase. *Hypertension*.

[90] Della Bruna R, Pinet F, Corvol P, Kurtz A (1991). Regulation of renin secretion and renin synthesis by second messenger in isolated mouse juxtaglomerular cells. *Cellular Physiology and Biochemistry*.

[91] Albinus M, Finkbeiner E, Sosath B, Osswald H (1998). Isolated superfused juxtaglomerular cells from rat kidney: a model for study of renin secretion. *American Journal of Physiology*.

[92] Kirchheim HR, Ehmke H, Hackenthal E, Lowe W, Persson P (1987). Autoregulation of renal blood flow, glomerular filtration rate and renin release in conscious dogs. *Pflügers Archiv European Journal of Physiology*.

[93] Itoh S, Carretero OA, Murray RD (1985). Renin release from isolated afferent arterioles. *Kidney International*.

[94] Ito S, Carretero OA, Abe K, Juncos LA, Yoshinaga K (1992). Macula densa control of renin release and glomerular hemodynamics. *Tohoku Journal of Experimental Medicine*.

[95] Ogawa K, Yamasato M, Taniguchi K (1995). Exocytosis of secretory granules in the juxtaglomerular granular cells of kidneys. *Anatomical Record*.

[96] Skøtt O, Taugner R (1987). Effects of extracellular osmolality on renin release and on the ultrastructure of the juxtaglomerular epithelioid cell granules. *Cell and Tissue Research*.

[97] Carey RM, Geary KM, Hunt MK (1990). Identification of individual renocortical cells that secrete renin. *American Journal of Physiology*.

[98] Hunt MK, Ramos SP, Geary KM (1992). Colocalization and release of angiotensin and renin in renal cortical cells. *American Journal of Physiology*.

[99] Ris H, Malecki M (1993). High-resolution field emission scanning electron microscope imaging of internal cell structures after Epon extraction from sections: a new approach to correlative ultrastructural and immunocytochemical studies. *Journal of Structural Biology*.

[100] Watanabe I, Koriyama Y, Yamada E (1992). High-resolution scanning electron microscopic study of the mouse submandibular salivary gland. *Acta Anatomica*.

[101] Everett AD, Carey RM, Chevalier RL, Peach MJ, Gomez RA (1990). Renin release and gene expression in intact rat kidney microvessels and single cells. *The Journal of Clinical Investigation*.

[102] Kendall ME, Hymer WC (1987). Cell blotting: a new approach to quantify hormone secretion from individual rat pituitary cells. *Endocrinology*.

[103] Kusaka Y, Kelly RA, Williams GH, Kifor I (2000). Coronary microvascular endothelial cells cosecrete angiotensin II and endothelin-1 via a regulated pathway. *American Journal of Physiology*.

[104] Kirk KL, Bell PD, Barfuss DW, Ribadeneira M (1985). Direct visualization of the isolated and perfused macula densa. *American Journal of Physiology*.

[105] Inoué S, Spring KR (1997). *Video Microscopy: The Fundamentals*.

[106] Ishihara Y, Sakurai T, Kimura T, Terakawa S (2000). Exocytosis and movement of zymogen granules observed by VEC-DIC microscopy in the pancreatic tissue en bloc. *American Journal of Physiology*.

[107] Tao C, Yamamoto M, Mieno H, Inoue M, Masujima T, Kajiyama G (1998). Pepsinogen secretion: coupling of exocytosis visualized by video microscopy and [Ca^2+^]_i_ in single cells. *American Journal of Physiology*.

[108] Zenisek D, Steyer JA, Almers W (2000). Transport, capture and exocytosis of single synaptic vesicles at active zones. *Nature*.

[109] Steyer JA, Horstmann H, Almers W (1997). Transport, docking and exocytosis of single secretory granules in live chromaffin cells. *Nature*.

[110] Ohara-Imaizumi M, Nakamichi Y, Tanaka T, Ishida H, Nagamatsu S (2002). Imaging exocytosis of single insulin secretory granules with evanescent wave microscopy: distinct behavior of granule motion in biphasic insulin release. *The Journal of Biological Chemistry*.

[111] Ma L, Bindokast VP, Kuznetsov A (2004). Direct imaging shows that insulin granule exocytosis occurs by complete vesicle fusion. *Proceedings of the National Academy of Sciences of the United States of America*.

[112] Wiegand UK, Duncan RR, Greaves J, Chow RH, Shipston MJ, Apps OK (2003). Red, yellow, green go!—a novel tool for microscopic segregation of secretory vesicle pools according to their age. *Biochemical Society Transactions*.

[113] Tokunaga M, Imamoto N, Sakata-Sogawa K (2008). Highly inclined thin illumination enables clear single-molecule imaging in cells. *Nature Methods*.

[114] Skøtt O (1986). Episodic release of renin from single isolated superfused rat afferent arterioles. *Pflügers Archiv European Journal of Physiology*.

[115] Lykkegard S, Poulsen K (1976). Ultramicroassay for plasma renin concentration in the rat using the antibody trapping technique. *Analytical Biochemistry*.

[116] Friis UG, Jensen BL, Jørgensen F, Andreasen D, Skøtt O (2005). Electrophysiology of the renin-producing juxtaglomerular cells. *Nephrology Dialysis Transplantation*.

[117] Toma I, Kang JJ, Peti-Peterdi J (2006). Imaging renin content and release in the living kidney. *Nephron Physiology*.

[118] Rosenblum WI, Nelson GH, Shimizu T (1992). L-Arginine suffusion restores response to acetylcholine in brain arterioles with damaged endothelium. *American Journal of Physiology*.

[119] Rosenblum WI, Nelson GH (1996). Singlet oxygen scavengers affect laser-dye impairment of endothelium-dependent responses of brain arterioles. *American Journal of Physiology*.

[120] Knight MM, Roberts SR, Lee DA, Bader DL (2003). Live cell imaging using confocal microscopy induces intracellular calcium transients and cell death. *American Journal of Physiology*.

[121] Hilgers KF, Veelken R, Müller DN (2001). Renin uptake by the endothelium mediates vascular angiotensin formation. *Hypertension*.

[122] Gomez RA, Lynch KR, Sturgill BC (1989). Distribution of renin mRNA and its protein in the developing kidney. *American Journal of Physiology*.

[123] Bosse HM, Böhm R, Resch S, Bachmann S (1995). Parallel regulation of constitutive NO synthase and renin at JGA of rat kidney under various stimuli. *American Journal of Physiology*.

[124] Wise PM, Scarbrough K, Larson G (1993). Assessment of gene expression and peptide secretion from individual cells. *Microscopy Research and Technique*.

[125] Lambolez B, Audinat E, Bochet P, Crepel F, Rossier J (1992). AMPA receptor subunits expressed by single Purkinje cells. *Neuron*.

[126] Schröppel B, Huber S, Horster M, Schlöndorff D, Kretzler M (1998). Analysis of mouse glomerular podocyte mRNA by single-cell reverse transcription-polymerase chain reaction. *Kidney International*.

[127] Meer DP, Eddinger TJ (1996). Heterogeneity of smooth muscle myosin heavy chain expression at the single cell level. *American Journal of Physiology*.

[128] Huber S, Schröppel B, Kretzler M, Schlöndorff D, Horster M (1998). Single cell RT-PCR analysis of ClC-2 mRNA expression in ureteric bud tip. *American Journal of Physiology*.

[129] Nuovo GJ (1992). *PCR In Situ Hybridization Protocols and Applications*.

[130] Teng B-Q, Murthy KS, Kuemmerle JF (1998). Expression of endothelial nitric oxide synthase in human and rabbit gastrointestinal smooth muscle cells. *American Journal of Physiology*.

[131] Block SM (2000). Making light work with optical tweezers. *Nature*.

[132] Kohda Y, Murakami H, Moe OW, Star RA (2000). Analysis of segmental renal gene expression by laser capture microdissection. *Kidney International*.

[133] Murakami H, Liotta L, Star RA (2000). IF-LCM: laser capture microdissection of immunofluorescently defined cells for mRNA analysis. *Kidney International*.

[134] Nagasawa Y, Takenaka M, Matsuoka Y, Imai E, Hori M (2000). Quantitation of mRNA expression in glomeruli using laser-manipulated microdissection and laser pressure catapulting. *Kidney International*.

[135] Sequeira López MLS, Cherñavvsky DR, Nomasa T, Wall L, Yanagisawa M, Gomez RA (2003). The embryo makes red blood cell progenitors in every tissue simultaneously with blood vessel morphogenesis. *American Journal of Physiology*.

[136] Jones CA, Hurley MI, Black TA (2000). Expression of a renin/GFP transgene in mouse embryonic, extra-embryonic, and adult tissues. *Physiological Genomics*.

[137] Taugner R, Nobiling R, Metz R, Taugner F, Bührle C, Hackenthal E (1988). Hypothetical interpretation of the calcium paradox in renin secretion. *Cell and Tissue Research*.

